# Portal vein recanalization without TIPS via a submillimeter cavernomatous channel in non-cirrhotic extrahepatic portal vein obstruction

**DOI:** 10.1186/s42155-026-00677-9

**Published:** 2026-04-09

**Authors:** Masayoshi Yamamoto, Takaki Hirano, Masaki Terunuma, Hiroshi Kondo

**Affiliations:** Department of Radiology, Teikyo University School of Medicine, 2-11-1 Kaga, Itabashi-Ku, Tokyo, Japan

To the Editor,

Physiological portal vein recanalization (PVR) without transjugular intrahepatic portosystemic shunt (TIPS) is increasingly recognized as a treatment option for selected non-cirrhotic patients with extrahepatic portal vein obstruction (EHPVO). When a traversable cavernomatous channel is present, restoration of hepatopetal flow without permanent portosystemic shunting may be feasible [1–3]. Marra et al. highlighted that physiological PVR without TIPS should be prioritized whenever technically achievable in non-cirrhotic EHPVO [2]. We report a technically challenging case in which PVR without TIPS was accomplished through an extremely fine, submillimeter cavernomatous channel that was not appreciated on routine imaging.

## Case description

**Fig. 1:**
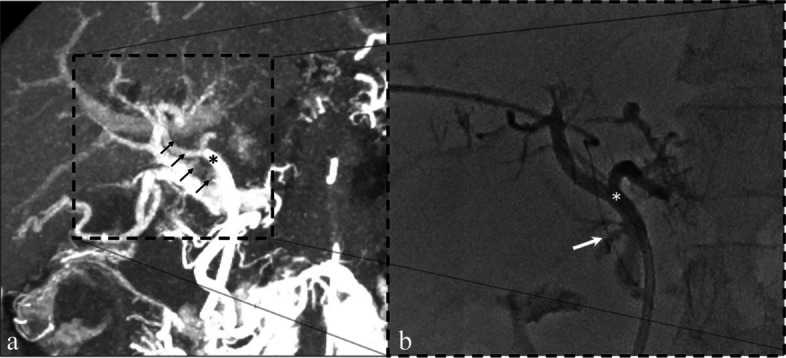
Identification and traversal of a submillimeter cavernomatous collateral. **a** CT during catheter-directed superior mesenteric arteriography with maximum intensity projection (MIP). The superior mesenteric vein (SMV) is strongly opacified, whereas the intrahepatic portal vein is faintly visualized. An arrow indicates a faint linear structure corresponding to a traversable submillimeter cavernomatous collateral connecting the SMV and an intrahepatic portal branch, which was not appreciated on routine portal venous phase CT. Transhepatic portography obtained through a 1.7-Fr microcatheter (Progreat λ, Terumo Corporation, Tokyo, Japan) advanced from the intrahepatic portal vein into the SMV via the same pathway (arrow), confirming successful intraluminal traversal. Asterisks (*) indicate corresponding distal SMV locations in panels **a** and **b**

**Fig. 2:**
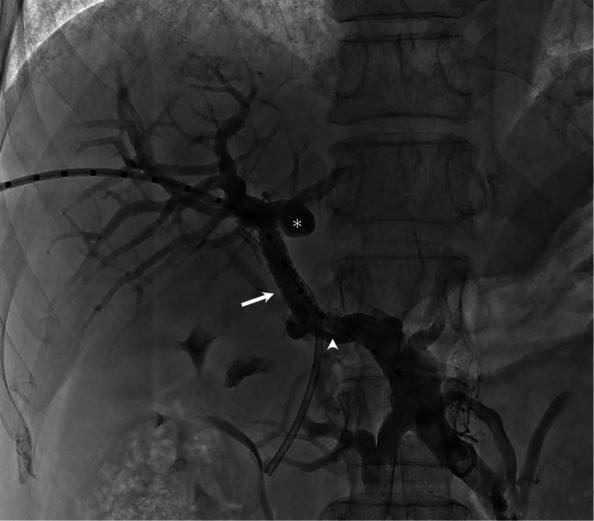
Portal vein reconstruction after recanalization. Superior mesenteric vein portography after portal vein reconstruction. A 7 × 39 mm balloon-expandable covered stent (Viabahn VBX, W. L. Gore & Associates, Flagstaff, AZ, USA) was deployed across the recanalized tract to provide immediate sealing, followed by a 10 × 80 mm self-expanding bare metal stent (Epic, Boston Scientific, Marlborough, MA, USA) to enlarge and stabilize the reconstructed portal vein. The asterisk (*) marks the stump of the native portal vein, and the arrowhead indicates adjacent thrombus. Restoration of hepatopetal flow was achieved without permanent portosystemic shunting, consistent with the physiological goals of portal vein recanalization alone in selected non-cirrhotic EHPVO patients [2, 3]

A 56-year-old man with long-standing non-cirrhotic EHPVO presented with recurrent duodenal variceal bleeding despite multiple endoscopic treatments, including endoscopic injection of N-butyl-2-cyanoacrylate. Non-target embolization of cyanoacrylate cement into the superior mesenteric vein (SMV) was suspected to have contributed to the progression of portal venous thrombosis, limiting further endoscopic options. Contrast-enhanced CT demonstrated chronic extrahepatic portal vein occlusion with cavernous transformation and portal biliopathy; however, direct continuity between the SMV and intrahepatic portal branches was not visualized on routine portal venous phase imaging (Figs. 1 and 2).

CT during catheter-directed superior mesenteric arteriography with maximum intensity projection (MIP) demonstrated a faint collateral channel connecting the SMV to an intrahepatic portal branch. The channel was estimated to be submillimeter in diameter and was not appreciated on routine portal venous phase CT. Under ultrasound and fluoroscopic guidance, an intrahepatic portal branch was punctured transhepatically, and careful micro-guidewire manipulation enabled successful traversal of the collateral into the SMV. Portography confirmed intraluminal access.

Given the extremely small caliber of the tract and the potential risk of extravasation during tract dilatation, a balloon-expandable covered stent was deployed to provide immediate sealing, followed by a self-expanding bare metal stent to enlarge and stabilize the reconstructed portal vein. The pressure gradient between the SMV and the intrahepatic portal vein decreased from 17 to 5 mmHg. No periprocedural complications occurred. Follow-up imaging at 3 months confirmed stent patency, and the patient remained free from rebleeding.

## Discussion

Most reports of PVR without TIPS in non-cirrhotic EHPVO involve cavernomatous channels that are identifiable on standard CT or conventional portography [1, 3]. In contrast, the present case demonstrates that physiological PVR may be feasible even when the only available pathway is a submillimeter collateral visible only on catheter-directed imaging. Recent technical reports describing sharp or electrified recanalization techniques highlight ongoing efforts to address complex chronic portal venous occlusions [4, 5]; however, these approaches differ conceptually from the present case, which relied on identifying and exploiting an extremely fine pre-existing physiological channel. The use of a covered stent was not intended as a general requirement for PVR without TIPS, but it was considered advantageous in this specific anatomy to provide immediate tract sealing during recanalization.

In the present case, the traversable pathway was not visualized as a discrete vessel on routine portal venous phase CT but appeared only as a faint linear structure on catheter-directed CT obtained during superior mesenteric arteriography. Although such subtle findings may be easily overlooked or regarded as nonspecific, careful interpretation in the appropriate hemodynamic context allowed recognition of a physiologically relevant cavernomatous collateral. This case highlights that targeted angiographic CT can reveal actionable portal venous pathways that are radiologically occult on standard imaging, thereby expanding the feasibility of physiological portal vein recanalization without TIPS in selected patients with chronic EHPVO.

## Data Availability

All data generated or analyzed during this study is included in this published article.

## Abbreviations

EHPVO: Extrahepatic portal vein obstruction
PVR: Portal vein recanalization
SMV: Superior mesenteric vein
TIPS: Transjugular intrahepatic portosystemic shunt
